# The effect of extracorporeal shock wave on joint capsule fibrosis based on A_2_AR-Nrf2/HO-1 pathway in a rat extending knee immobilization model

**DOI:** 10.1186/s13018-023-04420-1

**Published:** 2023-12-06

**Authors:** Hai Yuan, Kui Wang, Quan-Bing Zhang, Feng Wang, Yun Zhou

**Affiliations:** 1 Department of Rehabilitation Medicine, The Second Affiliated Hospital of Anhui Medical University, No. 678 Furong Road, Economic and Technological Development Zone, Hefei, 230601 China; 2 Research Center for Translational Medicine, The Second Affiliated Hospital of Anhui Medical University, Hefei, China; 3 Department of Rehabilitation Medicine, The Second People’s Hospital of Hefei City, Hefei, China

**Keywords:** Joint capsule fibrosis, Extracorporeal shock wave therapy, Adenosine A_2_A receptor, Neurotrophic factor e2-related factor 2, Heme oxidase-1

## Abstract

Joint capsule fibrosis, a common complication of joint immobilization, is mainly characterized by abnormal collagen deposition. The present study aimed to investigate the effect of extracorporeal shock wave therapy (ESWT) on reduced collagen deposition in the joint capsule during immobilization-induced joint capsule fibrosis. Additionally, the potential involvement of the adenosine A_2_A receptor (A_2_AR)-Neurotrophic factor e2-related factor 2 (Nrf2)/Haem oxygenase-1 (HO-1) pathway was explored. Thirty 3-month-old male Sprague–Dawley rats were randomly assigned to five groups: control (C), immobilization model (IM), natural recovery (NR), ESWT intervention (EI), and ESWT combined with A_2_AR antagonist SCH 58261 intervention (CI). After the left knee joints of rats in the IM, NR, EI and CI groups were immobilized using a full-extension fixation brace for 4 weeks, the EI and CI groups received ESWT twice a week for 4 weeks. The CI group was also treated with ESWT following intraperitoneal injection of SCH 58261 (0.01 mg/kg) for 4 weeks. The range of motion of the left knee joint was measured, and the protein levels of collagens I and III, A_2_AR, phosphorylated-protein kinase A/protein kinase A (p-PKA/PKA), p-Nrf2/Nrf2, and HO-1 were analysed by Western blotting. The IM and NR groups showed significantly greater arthrogenic contracture than the C group (*P* < 0.05). Compared to the NR group, the EI and CI groups exhibited significant improvement in arthrogenic contracture (*P* < 0.05). Conversely, the EI group showed lower contracture than the CI group (*P* < 0.05). Similar results were observed for collagen deposition and the protein levels of collagens I and III. The intervention groups (EI and CI groups) showed higher levels of p-Nrf2/Nrf2 and HO-1 than the NR group (*P* < 0.05). Moreover, the EI group exhibited higher levels of p-PKA/PKA, p-Nrf2/Nrf2, and HO-1 than the CI group (*P* < 0.05). However, no significant difference was found in the A_2_AR levels among the five groups (*P* > 0.05). ESWT may activate A_2_AR, leading to the phosphorylation of PKA. Subsequently, Nrf2 may be activated, resulting in the upregulation of HO-1, which then reduces collagen deposition and alleviates immobilization-induced joint capsule fibrosis.

## Background

Immobilization is commonly used to protect injured joints. However, prolonged immobilization can lead to joint contracture, which decreases joint function and lowers the quality of life of patients [1, 2]. Joint contracture is a frequently observed complication resulting from joint immobilization, with arthrogenic factors playing an important role in its development over prolonged periods of immobilization [3]. Addressing arthrogenic contracture during clinical joint rehabilitation is crucial for restoring joint function [4]. Pathological changes associated with immobilization-induced arthrogenic contracture, such as joint capsule fibrosis, collagen deposition, and synovial hypertrophy, have been observed to contribute to a limited range of motion [5, 6]. Among these changes, joint capsule fibrosis is widely acknowledged as the primary determining factor. It is characterized by inflammation, collagen deposition and fibroblast proliferation [4, 7]. Therefore, it is imperative to promptly investigate joint capsule fibrosis linked to joint contracture and explore potential treatment options.

As the main stress-bearing joint of the lower limbs, the knee joint is prone to injury during sports activities [8]. Extended immobilization is a commonly used method to protect injured knees and maintain them in a neutral position. In line with clinical practice, an extended knee immobilization model has been used for studying joint contractures [9, 10]. Zhang QB et al. utilized this model to examine the impact of electrical stimulation on knee joint contracture [11]. Rats were immobilized in the fully extended position of the left knee joint for 4 weeks to induce joint capsule fibrosis, and these results showed an increase in total cell count and collagen deposition in the joint capsule [12]. In another study, an increase in collagen proliferation was observed in the joint capsule after extended knee fixation for 4 weeks [13]. Therefore, the extended knee immobilization model mimics the clinical manifestations of extended immobilization in humans and can be used to study knee joint capsule fibrosis induced by immobilization.

Fibrosis and inflammation have been recognized as critical factors in the development of joint capsule fibrosis [14, 15]. Immobilization for 4 days induces inflammatory changes in the joint capsule, marked by synovial histological alterations and mononuclear cell infiltration [16]. Elevated levels of proinflammatory cytokines, such as tumour necrosis factor-alpha (TNF-α) [17], interleukin-1beta (IL-1β), and IL-6 [18, 19], have also been observed in immobilized rat knee joints.

The inflammation of the joint capsule may be induced by immobilization, suggesting that inflammation could be a marker of joint contracture [20]. Adenosine is an endogenous modulator that maintains cellular and tissue homeostasis. It interacts with G protein-linked receptor subtypes, such as adenosine A_2_A receptor (A_2_AR), to regulate cyclic AMP levels [21]. Stressful stimulation can increase the rate of conversion of intracellular adenosine triphosphate (ATP) to adenosine. A_2_AR exerts anti-inflammatory effects and is dynamically mediated by macrophages and proinflammatory cytokines [22]. Previous studies have shown that activated A_2_AR has an antifibrotic effect on cardiac fibroblasts [23, 24]. Another study showed that activation of A_2_AR reduces myocardial fibrosis by suppressing the levels of collagens I and III [25]. However, the specific anti-inflammatory role of adenosine and its receptors in joint capsule fibrosis has yet to be elucidated.

Haem oxygenase-1 (HO-1) is an essential enzyme responsible for breaking down heme into anti-inflammatory byproducts, including carbon monoxide (CO), biliverdin, and iron ions (Fe^2+^) [26, 27]. The neurotrophic factor e2-related factor 2 (Nrf2)/HO-1 signalling pathway is critical in the body’s inflammatory response [28]. The activation of Nrf2 upregulates the expression of HO-1, which then inhibits the production of proinflammatory factors [29, 30]. The use of inhaled CO in acute lung injury mice has shown an anti-inflammatory effect [31]. HO-1 gene transfer was found to be protective against lung fibrotic injury in a mouse lung fibrosis model [32]. This protective effect has been attributed to the anti-proliferative effects of CO. However, the involvement of Nrf2/HO-1 in the inflammatory response of joint capsule fibrosis has not been explored.

Extracorporeal shock wave therapy (ESWT) was previously used to treat renal calculi and has been currently applied to various orthopaedic conditions with promising results [33–36]. ESWT has been shown to reduce inflammation, release adhesions, and modulate fibroblast differentiation and apoptosis [37]. Our previous animal experiments have confirmed the biological effects of ESWT in decreasing joint capsule fibrosis to improve arthrogenic contracture [12, 13]. Some studies have shown that ESWT prevents joint capsule fibrosis in an immobilized knee model [38] and reduces collagen expression and fibronectin levels [39]. Nonetheless, the precise mechanism of how ESWT inhibits the hyperexpression of collagens and joint capsule fibrosis remains unclear.

Therefore, the present study aimed to explore the potential benefits of ESWT in treating knee joint capsule fibrosis and investigate the mechanisms underlying the efficiency of this treatment. Specifically, we focused on the A_2_AR-Nrf2/HO-1 pathway and its potential role in mediating the effects of ESWT on knee joint capsule fibrosis.

## Methods

**Fig. 1:**
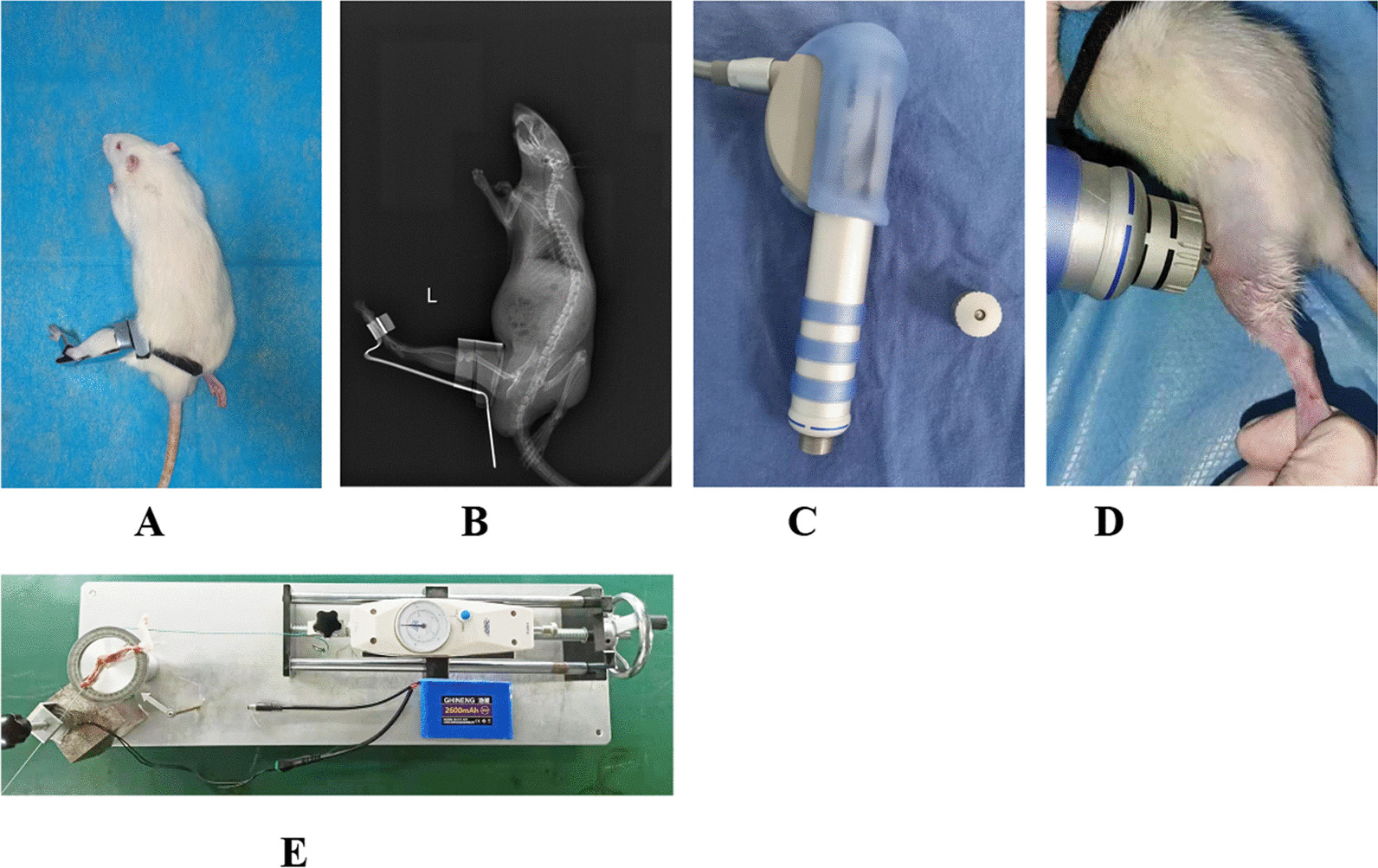
Schematic diagram illustrating the knee immobilized model, ESWT, and measurement of joint contracture. **A** The immobilized model of left knee joint. **B** The X-ray result of the model in the left knee joint. **C** Extracorporeal Shock Wave and the 6 mm diameter warhead. **D** ESWT. **E** Assessment of the range of motion of the left knee joint following myotomy

Thirty 3-month-old male Sprague–Dawley rats weighing 250–300 g were obtained from the Experimental Animal Center at Anhui Medical University, Hefei, China. The rats were housed in cages under controlled conditions at 24–25 °C with 12-h light/dark cycle and provided standard food and water. The rats were randomly and equally assigned to five groups: control (C), immobilization model (IM), natural recovery (NR), extracorporeal shock wave therapy intervention (EI), and extracorporeal shock wave therapy combined with A_2_AR antagonist SCH 58261 intervention (CI). Anaesthesia was induced in all rats in the IM, NR, EI, and CI groups by intraperitoneal injection with 2% sodium pentobarbital sodium (40 mg/kg), after which their left knee joints were immobilized using a full-extension fixation tool for 4 weeks (Patent No.202120470158.0), as described previously (Fig. 1A, B) [12, 13, 40].

The safety and effectiveness of the immobilized model were monitored daily. Rats in the C group were euthanized with an overdose of sodium pentobarbital (150 mg/kg) after 8 weeks of unrestricted movement within the cage. The IM group was euthanized immediately after removing the external fixation brace. The rats in the NR group were euthanized after 4 weeks of free movement in the cage following brace removal. The EI group received ESWT intervention for 4 weeks after brace removal, while the CI group underwent ESWT after intraperitoneal injection of SCH 58261 (0.01 mg/kg) for 4 weeks [41] (Fig. 1C, D).

The EI and CI groups were treated with ESWT for 4 weeks, twice a week, using the Dolor Clast device from Switzerland. The hair around the left knee joint was cleaned before applying the ultrasound gel evenly to the skin around the patella using a 6-mm-diameter ESW warhead. Then, ESWT was administered at a common therapeutic dose (1.5 bar, 6 Hz, 2000 times) [13] (Fig. 1C, D).

Next, a mechanical measuring device was used to measure the joint’s range of motion (ROM), as described previously (Patent No. LZ202120996643.1) [12, 13]. The ROM of the left knee joint in each rat was measured after myotomy using a standard torque of 0.053 N m (Fig. 1E). We calculated the degree of arthrogenic contracture using the following formula: Arthrogenic contracture = ROM after myotomy (of the control knee)—ROM after myotomy (of the contracted knee) [12, 13].

After measuring the range of motion (ROM) of the left knee joint, the supra-patellar ligament was initially severed to isolate the anterior joint capsule. Subsequently, we carefully removed the chosen anterior joint capsule along the edge of the knee joint and patella, obtaining a complete sample. It is essential to remove as much surrounding adipose tissue as possible during the operation. Finally, one-third of the selected medial joint capsule in the knee was preserved in 4% paraformaldehyde at 4 °C overnight for histopathological examination, while the remaining two-thirds of the selected lateral tissue were snap-frozen in liquid nitrogen and stored at − 80 °C for Western blotting analysis. The biliverdin, TNF-α, IL-1β, and IL-6 levels in the tissues were measured on a microplate reader (Varioskan LUX, Thermo Fisher Scientific, USA) according to the ELISA protocol. It is worth mentioning that equal-sized specimens were used for both Western blotting and ELISA in each sample.

For histological analysis, the tissue specimens were dehydrated using graded alcohol and embedded in paraffin. Then, sagittal sections with a thickness of 6 μm were obtained using a microtome. Subsequently, the sections were deparaffinized and subjected to haematoxylin–eosin (H&E) and Masson staining, according to the manufacturer’s instructions, to examine the pathological changes, including the total cell count and collagen deposition in the joint capsule. Following staining, the sections were examined under a microscope (BX43F, OLYMPUS, Tokyo 163-0914, Japan) at a magnification of 400×, and three fields were randomly selected in the section. The number of cells in each field was counted, and the average value was then calculated. Finally, the cell number was converted to a number per mm^2^ joint capsule. To assess collagen deposition, the percentage of the blue-stained area in the joint capsule tissue was measured using Image-Pro Plus 6.0 software.

The protein levels of A_2_AR, p-PKA/PKA, p-Nrf2/Nrf2, HO-1, collagens I and III, and β-tubulin were analysed by Western blotting. Briefly, tissue samples were ground to a fine powder using liquid nitrogen and homogenized with RIPA lysis buffer (Yamei, China). After centrifugation, the supernatant containing the proteins was collected. An equivalent amount of protein was separated by an SDS-PAGE Kit (Yamei, China) and transferred to polyvinylidene fluoride microporous membranes by electrotransfer. The membranes were then blocked with Protein Free Rapid Blocking Buffer (1×) at room temperature for 10 min. Subsequently, the membranes were washed three times with TBST for 10 min each before probing with the primary antibodies at 4 °C overnight [rabbit anti-A_2_AR antibody (1:2000; Affinity, America), rabbit anti-p-PKA/PKA antibody (1:2000; Affinity, America), rabbit anti-p-Nrf2/Nrf2 antibody (p-Nrf2, 1:2000; Nrf2, 1:1000; Affinity, America), rabbit anti-HO-1 antibody (1:5000; Affinity, America), rabbit anti-collagen I or III antibody (1:1000; Affinity, America), and rabbit anti-β-tubulin antibody (1:10,000; Affinity, America)], followed by incubation with peroxidase-conjugated affinity-purified goat anti-rabbit IgG-HRP (1:100,000; Affinity, America)at room temperature for 90 min.

Finally, the membranes were developed using an enhanced chemiluminescent (ECL) substrate was added and the target signal was captured on digital imaging equipment (UVP ChemStudio 515, Analytik Jena AG, Germany). The density of each band was quantified using Image-Pro Plus 6.0 software. The protein levels of A_2_AR, p-PKA/PKA, p-Nrf2/Nrf2, HO-1, and collagen I or III were normalized to those of β-tubulin, which served as the loading control.

The data are presented as the mean ± standard deviation and were analysed using SPSS 26.0. The differences between groups were compared using one-way ANOVA with the SNK post hoc test. Various factors, including arthrogenic contracture, degree of collagen deposition, and the expression levels of A_2_AR, p-PKA/PKA, p-Nrf2/Nrf2, HO-1, biliverdin, and collagen I or III, were compared across the groups. *P* < 0.05 indicated a statistically significant difference.

## Results

### Arthrogenic contracture

The degree of arthrogenic contracture in each group is presented in Table 1. The IM and NR groups exhibited significantly greater arthrogenic contracture than the C group (*P* < 0.05). In contrast, both the EI and CI groups showed a significant improvement in arthrogenic contracture compared to that in NR group (*P* < 0.05), indicating a positive impact of ESWT on reducing the degree of joint contracture. Additionally, the degree of contracture was found to be smaller in the EI group than in the CI group (*P* < 0.05).

**Table 1:** The degree of arthrogenic contracture (mean ± standard deviation)

Group	Number	Degree of arthrogenic contracture (°)
C	6	0.0 ± 0.0
IM	6	41.1 ± 4.3*
NR	6	45.8 ± 4.8*
EI	6	30.3 ± 5.0*^#%^
CI	6	39.0 ± 3.6*^%&^

*C* Control group, *IM* Immobilization model group, *NR* Natural recovery group, *EI* Extracorporeal shock wave intervention group, *CI* Extracorporeal shock wave combined with A_2_AR antagonist SCH 58261 intervention

*P < 0.05 versus the C group

^#^*P* < 0.05 versus the IM group

^%^*P* < 0.05 versus the NR group

^&^*P* < 0.05 versus the EI group

### Cell count

**Fig. 2:**
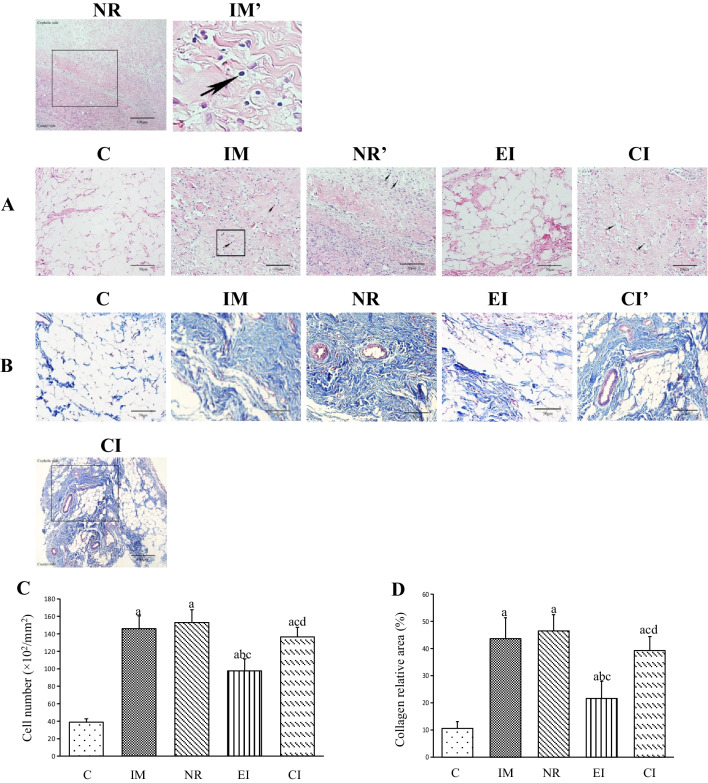
H&E and Masson staining. **A** H&E staining (The boxed area in field NR at a magnification of 200 × is highlighted in field NR’. The boxed area in field IM is highlighted in field IM’); **B** Masson staining (The boxed area in field CI at a magnification of 200 × is highlighted in field CI’); **C** Quantitative analysis of the total number of cells (The values are the mean ± standard deviation); **D** Quantitative analysis of the percentage of collagen deposition (blue area) (The values are the mean ± standard deviation). a *P* < 0.05 versus the C group, b *P* < 0.05 versus the IM group, c *P* < 0.05 versus the NR group, d *P* < 0.05 versus the EI group. *C* Control group, *IM* Immobilization model group, *NR* Natural recovery group, *EI* Extracorporeal shock wave intervention group, *CI* Extracorporeal shock wave combined with A_2_AR antagonist SCH 58261 intervention. The Arrow: the inflammatory cell

The results of H&E staining and quantitative analysis are presented in Figs. 2A, C, respectively. In the present study, the number of cells in the IM and NR groups was higher than that in the C group (*P* < 0.05), while both groups showed a marked increase in spindle-forming fibroblast-like cells, in addition to inflammatory cell infiltration.

Furthermore, the total number of cells in the EI group significantly decreased compared to that in the NR group (*P* < 0.05). Moreover, the total number of cells was significantly lower in the EI group than in the CI group (*P* < 0.05).

### Collagen deposition and expression of collagens I and III levels

**Fig. 3:**
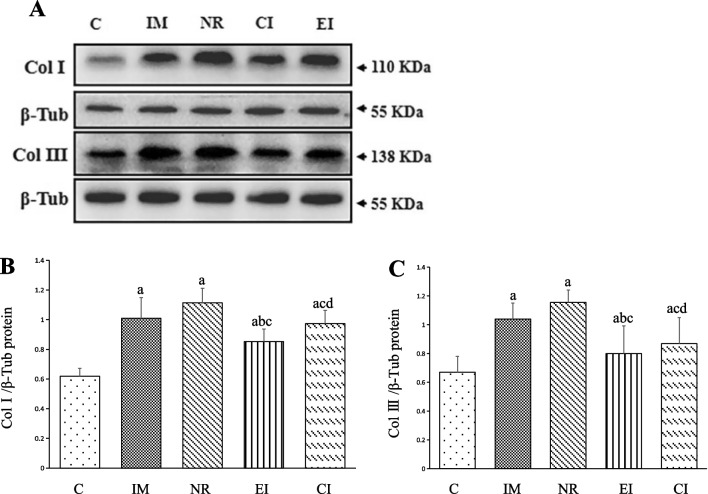
Protein expression levels of Collagens I and III relative to β-Tubulin (The values are the mean ± standard deviation). **A** Western blotting bands of Collagens I and III, and β-Tubulin proteins; **B** The quantitative analysis of Collagen I; **C** The quantitative analysis of Collagen III. a *P* < 0.05 versus the C group, b *P* < 0.05 versus the IM group, c *P* < 0.05 versus the NR group, d *P* < 0.05 versus the EI group. *C* Control group, *IM* Immobilization model group, *NR* Natural recovery group, *EI* Extracorporeal shock wave intervention group, *CI* Extracorporeal shock wave combined with A_2_AR antagonist SCH 58261 intervention. *Col I* Collagen I, *Col III* Collagen III, *β-Tub* β-Tubulin

The results of Masson staining are presented in Fig. 2B, D, respectively. The IM and NR groups exhibited higher collagen deposition than the C group (*P* < 0.05). The intervention groups (EI and CI groups) showed a significant decrease in collagen deposition compared to that in the NR group (*P* < 0.05). Moreover, the CI group had a more collagen deposition than the EI group (*P* < 0.05). Similar results were observed for the protein levels of collagens I and III (Fig. 3A–C).

### Expression of protein levels of A_2_AR, p-PKA/PKA, p-Nrf2/Nrf2, and HO-1

**Fig. 4:**
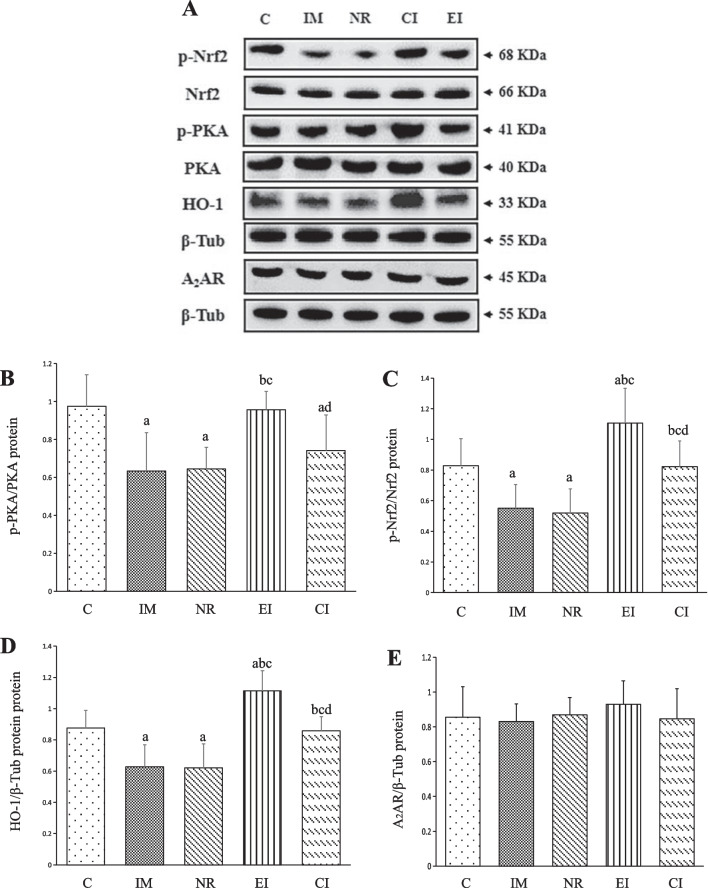
Protein expression levels of p-PKA/PKA, p-Nrf2/Nrf2, HO-1, A_2_AR and relative to β-Tubulin (The values are the mean ± standard deviation). **A** Western blotting bands of p-PKA/PKA, p-Nrf2/Nrf2, HO-1, A_2_AR and β-Tubulin proteins; **B** The quantitative analysis of p-PKA/PKA; **C** The quantitative analysis of p-Nrf2/Nrf2; **D** The quantitative analysis of HO-1; **E** The quantitative analysis of A_2_AR. a *P* < 0.05 versus the C group, b *P* < 0.05 versus the IM group, c *P* < 0.05 versus the NR group, d *P* < 0.05 versus the EI group. *C* Control group, *IM* Immobilization model group, *NR* Natural recovery group, *EI* Extracorporeal shock wave intervention group, *CI* Extracorporeal shock wave combined with A_2_AR antagonist SCH 58261 intervention. *PKA* Protein kinase A, *HO-1* Haem oxidase-1, *Nrf2* Neurotrophic factor e2-related factor 2, *A*_*2*_*AR* Adenosine A_2_A receptor, *β-Tub* β-Tubulin

The protein levels of A_2_AR, p-PKA/PKA, p-Nrf2/Nrf2, and HO-1 were analysed in the five groups of specimens, as shown in Fig. 4A–E. The IM and NR groups exhibited significantly lower levels of p-PKA/PKA, p-Nrf2/Nrf2, and HO-1 proteins than the C group (*P* < 0.05). However, the intervention groups (EI and CI groups) showed higher levels of p-Nrf2/Nrf2 and HO-1 proteins than the NR group. Furthermore, the levels of p-PKA/PKA, p-Nrf2/Nrf2, and HO-1 were significantly higher in the EI group than in the CI group (*P* < 0.05) (Fig. 4A–D). In contrast, no significant difference was observed in the level of A_2_AR among the five groups (*P* > 0.05) (Fig. 4A,E).

### Activation of HO-1

Biliverdin, produced by HO-1, serves as an indicator of HO-1 activity. In this study, the levels of biliverdin were significantly lower in the IM and NR groups than in the C group (*P* < 0.05) but were significantly higher in the EI and CI groups than in the NR group (*P* < 0.05). Notably, the EI group exhibited a significantly higher level of biliverdin than the CI group (*P* < 0.05) (Table 2).

**Table 2:** The level of biliverdin (mean ± standard deviation)

Group	Number	Biliverdin (pg/ml)
C	6	33.49 ± 2.80
IM	6	26.81 ± 3.09*
NR	6	24.66 ± 1.85*
EI	6	44.26 ± 7.23*^#%^
CI	6	36.08 ± 4.54^#%&^

*C* Control group, *IM* Immobilization model group, *NR* Natural recovery group, *EI* Extracorporeal shock wave intervention group, *CI* Extracorporeal shock wave combined with A_2_AR antagonist SCH 58261 intervention

*P < 0.05 versus the C group, ^#^*P* < 0.05 versus the IM group, ^%^*P* < 0.05 versus the NR group, ^&^*P* < 0.05 versus the EI group

### Levels of TNF-α, IL-1β, and IL-6

**Fig. 5:**
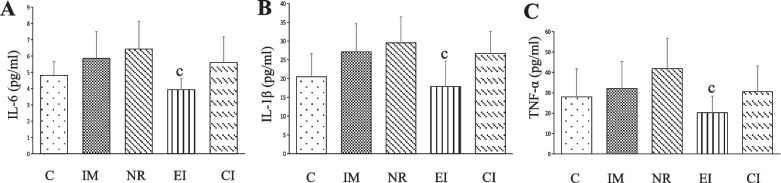
The levels of IL-6, IL-1β, and TNF-α among groups (The values are the mean ± standard deviation). **A** IL-6, **B** IL-1β, **C** TNF-α. c *P* < 0.05 versus the NR group. *TNF-α* Tumour necrosis factor-alpha, *IL-1β* Interleukin-1beta, *IL-6* Interleukin-6

Significant decreases in the levels of TNF-α, IL-1β, and IL-6 were found in the EI group compared to the NR group (*P* < 0.05). However, there were no statistically significant differences between the NR group and the C group (*P* > 0.05) (Fig. 5A–C).

## Discussion

Joint immobilization is commonly implemented to protect injured joints after fractures and ligament injuries [42, 43]. Nonetheless, it can lead to various side effects, including joint contracture and degeneration of the articular structure [44]. Strikingly, articular structure degeneration, such as joint capsule fibrosis, plays a central role in the immobilization-induced arthrogenic contracture [45]. An aluminium fixation brace was used to immobilize the knee joint in an extended position [40]. To reduce the brace fall-off, the ankle was fixed with plantar flexion. However, the fixed brace did not hinder the rats' free movement within the cage, as the hip joint was unfixed, which closely resembles the fixation of the knee joint in clinical settings. In the present study, the results showed a decrease in the ROM of the knee following 4 weeks of full-extension immobilization, indicating significant movement disorder of the knee in the immobilization-induced animal model. ESWT was applied in this study and was found to be beneficial to immobilization-induced joint contracture. However, when ESWT was combined with an adenosine A_2_AR antagonist, ROM was not altered significantly, but rather worsened. ESWT has a positive impact on reducing joint contracture, which may be attributed to the activation of A_2_AR.

Collagens I and III play a role in joint capsule fibrosis [13, 15]. After 4 weeks of immobilization, the levels of collagens I and III increased in the shoulder of rats [46]. Sasabe et al. also found that collagen deposition in the posterior capsule was increased during 4 weeks of immobilization [45]. Additionally, an increase in the collagens in connective tissue was confirmed [7, 13], which was consistent with our study that collagens I and III were increased after 4 weeks of full-extension immobilization, indicating excessive collagen deposition in the joint capsule by immobilization-induced joint capsule fibrosis. In addition, a previous study suggested that the excess buildup of collagens could contribute to the joint capsule fibrosis induced by full-extension immobilization [12]. Thus, the excessive deposition of collagens is a primary factor in the development of joint contracture and is associated with restricted joint movement. Moreover, the main approach to alleviate joint capsule fibrosis should focus on reducing the collagen deposition.

During the initial 2 weeks of joint fixation, an inflammatory response occurs in the joint capsule [47], which leads to arthrofibrosis, whereby neutrophils infiltrate the injury site, followed by macrophage migration and fibroblast attachment, ultimately resulting in collagen deposition [48, 49]. Several studies have reported a link between inflammation and fibrosis in various organs, including joints [50, 51]. ESWT has shown promising results in reducing fibrous tissue adhesion and fibrous cell density, restoring joint mobility, and preventing abnormal fibrous tissue formation [52]. Additionally, ESWT also downregulates type I collagen and connective tissue growth factor expression [53]. Low-energy ESWT has been found to reduce inflammation to inhibit fibroblast density via macrophage transfer [54]. The results of H&E and Masson staining and Western blotting showed that the cell count and collagen deposition significantly increased after immobilization. However, ESWT caused a decline in cell number and collagen deposition, which was consistent with the improved arthrogenic contracture. This phenomenon indicated a positive improvement of ESWT on immobilization-induced joint capsule fibrosis by decreasing the total cell number (fibroblasts and inflammatory cells) and collagen deposition in the joint capsule. However, the A_2_AR antagonist inhibited the effects of ESWT. ESWT has been found to exert various mechanical stress effects on different human tissues, which in turn promotes the release of joint and soft tissue adhesion and improves fibrosis. The application of ESWT might trigger the activation of A_2_AR in the joint capsule through mechanical and cavitation effects. Physiologically, ATP levels are low but increase rapidly in pathological conditions such as inflammation or apoptosis, where it is broken down into adenosine diphosphate and adenosine [55]. The biotherapeutic effect of ESWT is primarily achieved through mechanical transmission and the cavitation effect. Qi et al. discovered that ESWT treatment can increase the extracellular concentration of ATP [56], possibly due to the cavitation effect [57]. Another study suggested that therapeutic ultrasound, which utilizes the cavitation effect, can increase muscle perfusion by shear-dependent increases in the level of ATP, which can act through the diverse signalling pathways [58]. Thus, the molecular mechanisms by which ESWT reduces collagen deposition through A_2_AR need to be explored.

Adenosine plays a crucial role in anti-inflammatory processes by activating A_2_AR, which then inhibits the release of proinflammatory mediators [59]. A study has shown that activating A_2_AR inhibits the Wnt/β-catenin pathway and reduces collagen deposition in silica-induced lung fibrosis [60]. Additionally, A_2_AR may play a role in reducing fibrosis involved in cardiac pathological remodelling [61, 62] and pulmonary fibrosis through the Rap1 [63] or BMP7/Smad1/5 signalling pathway [64]. However, the protein expression of A_2_AR was not changed significantly among the five groups in our study, suggesting that the effect of ESWT on joint contracture may not be achieved by directly increasing the level of A_2_AR. However, it may exert its biological effects by activating A_2_AR. After A_2_AR activation, adenylate cyclase is stimulated to degrade intracellular ATP to produce cAMP, followed by PKA, which is activated by cAMP to phosphorylate a series of messenger molecules to exert biological effects [65, 66]. Interestingly, we also observed that the p-PKA expression levels were increased after ESWT but decreased significantly after the administration of an A_2_AR antagonist, indicating that ESWT upregulates p-PKA expression through A_2_AR activation. Thus, our findings suggested that the therapeutic effects of ESWT on joint contracture may be mediated through A_2_AR activation and the subsequent increase in p-PKA levels. Thus, the investigation is essential to understand the anti-inflammatory mechanisms by which ESWT treats joint capsule fibrosis and the role of A_2_AR. In addition, the activation of A_2_AR in the joint capsule has the potential to inhibit joint capsule fibrosis, making it a possible target for delaying or treating the development of joint contracture in clinical settings.

HO-1 is a crucial regulator of immune and inflammatory functions, owing to its anti-inflammatory properties [26, 27]. CO is one of its reaction products that has been recognized as a natural physiological regulator, influencing intracellular signalling pathways and suppressing inflammatory responses [67]. It can also regulate fibroblast proliferation, thereby protecting against organ fibrosis. Some studies have demonstrated that HO-1 overexpression prevents renal fibrosis during unilateral ureteral obstruction by inhibiting Wnt/β-catenin signalling [68] and MKK3-dependent pathways [69]. Similar antifibrotic effects of CO were also observed in pulmonary fibrosis [70]. Treatment with inhaled exogenous CO has been linked to a protective effect against pulmonary fibrosis in mice [71]. Biliverdin, a byproduct of HO-1, is an indirect indicator of CO production levels. In this study, the level of biliverdin was similar to that of HO-1, indicating that ESWT increases the p-PKA levels in the body by activating the A_2_AR receptor. These molecules increase the HO-1 level and generate CO to inhibit the inflammatory response and collagen deposition, ultimately alleviating arthrogenic contracture. Nrf2 is a transcription factor found in various cell types and is negatively regulated by Keap1 under normal conditions [72]. As a response to oxidative stress, Keap1 dissociates from and facilitates the translocation of Nrf2 to the nucleus and regulates the expression of genes such as HO-1 against oxidative stress and inflammation [73]. In the present study, ESWT increased p-PKA levels, activated Nrf2, upregulated HO-1 expression, and inhibited joint capsule fibrosis. However, when the A_2_AR antagonist was administered, p-PKA, p-Nrf2, and HO-1 levels decreased, collagen deposition increased, and joint capsule fibrosis worsened. These findings suggested that ESWT activates the A_2_AR receptor, increases p-PKA levels, activates Nrf2, upregulates HO-1 expression, and ultimately relieves joint capsule fibrosis. Moreover, additional in vitro experiments are needed to confirm the involvement of this pathway in inflammation-induced joint capsule fibrosis.

In addition, low levels of TNF-α, IL-1β, and IL-6 were found in the EI group, indicating a decrease in inflammation. However, no significant differences were found between the NR group and the C group. This observation may be attributed to the early stage of joint contracture formation characterized by inflammation [14–16], which gradually decreases in the later stage [20]. Moreover, Michelsson JE et al. reported the occurrence of synovitis during knee remobilization in rabbits after five weeks of immobilization [16]. These findings indicate that proinflammatory cytokines may play an important role in the development of joint capsule contracture. Therefore, in cases of joint contracture resulting from prolonged immobilization and decreased levels of proinflammatory cytokines in the EI group, suppressing the inflammatory response using ESWT may be a strategy to alleviate joint contracture.

## Conclusions

In conclusion, this study provides evidence that ESWT reduces the total number of cells and collagen deposition in the joint capsule, thereby improving joint capsule fibrosis caused by long-term immobilization. This effect is achieved by activating A_2_AR and increasing the level of p-PKA, which in turn triggers the Nrf2/HO-1 pathway. As a result, the expression of collagens I and III in the joint capsule is downregulated, ultimately alleviating joint capsule fibrosis and arthrogenic contracture. These findings highlight the therapeutic potential of ESWT in the management of joint contracture and offer valuable insights into the underlying mechanism of action.

## Limitations

The present study has several limitations that need to be addressed. First, the PKA/MAPK signalling system has been reported [74]. Additionally, Yen TL et al. found that the MAPK-Nrf2-HO-1 signalling cascade can provide protective effects against ischaemic stroke in rats. Nrf2 phosphorylation and nuclear translocation were observed, and these activities were inhibited by a p38 MAPK inhibitor (SB203580) [75]. Therefore, the role of the A_2_AR-PKA-MAPK-Nrf2-HO-1 pathway should be explored further, as it may play a key role in cellular and molecular mechanisms of joint contracture. Second, although no statistical significance was found in the p-Nrf2 and HO-1 levels between the CI and C groups, a notable difference was observed between the collagen I/III levels of these two groups. This discrepancy might be attributed to ESWT inhibiting collagen deposition through other pathways. Hu C et al. reported that the binding of TGF-β to its receptor can activate the Smad protein, which subsequently transmits signals to the nucleus. ESWT treatment has the potential to inhibit the excessive activation of the TGF-β1/Smad2/3/JNK pathway [13], thus preventing joint capsule fibrosis and alleviating joint contracture. Additionally, the MAPK/ERK pathway may also be involved [12].

## Data Availability

The data that support the findings of this study are available on request from the corresponding author.

## Abbreviations

ESWT: Extracorporeal shock wave therapy
A_2_AR: Adenosine A_2_A receptor
Nrf2: Neurotrophic factor e2-related factor 2
HO-1: Haem oxidase-1
CO: Carbon monoxide
PKA: Protein kinase A
